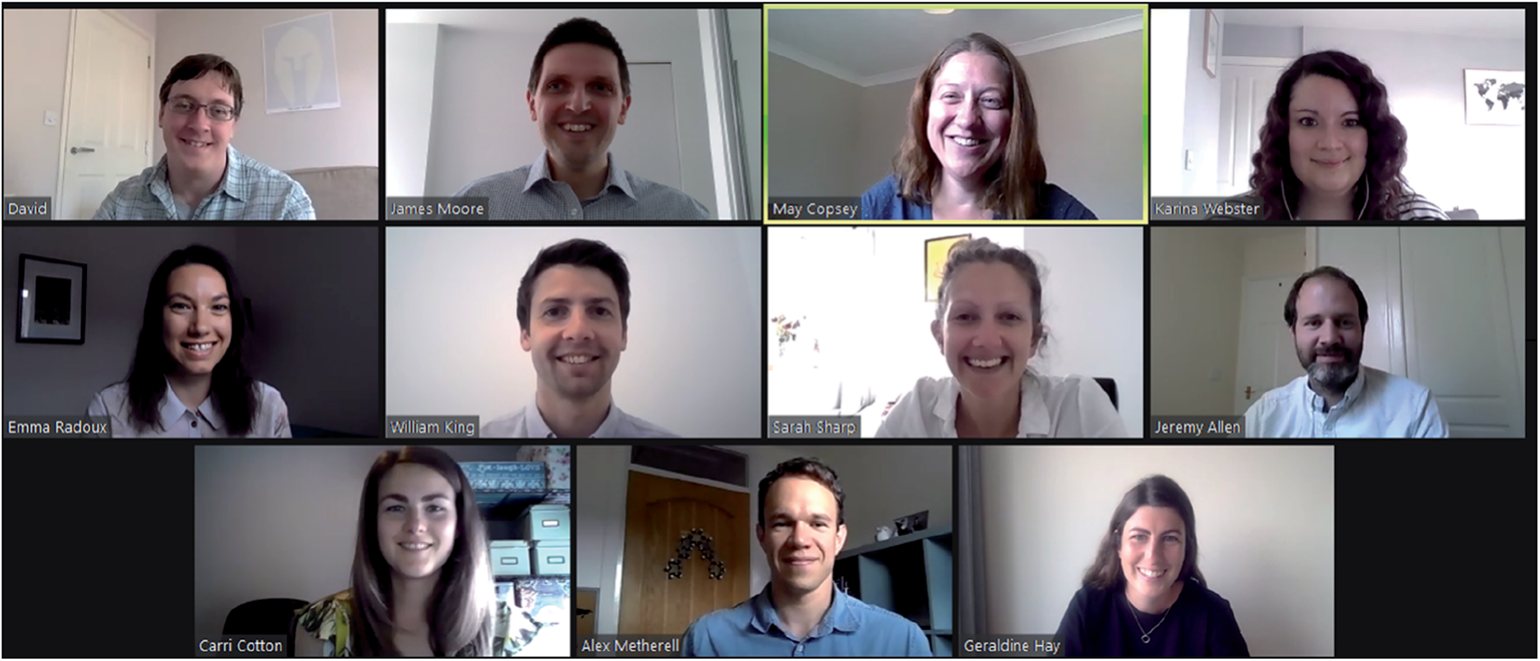# Celebrating 10 years of *Chemical Science*

**DOI:** 10.1039/d0sc90127j

**Published:** 2020-06-30

## Abstract

Welcome to the first of our special anniversary issues planned for this year, marking 10 years since *Chemical Science* published its first issue, back in July 2010.
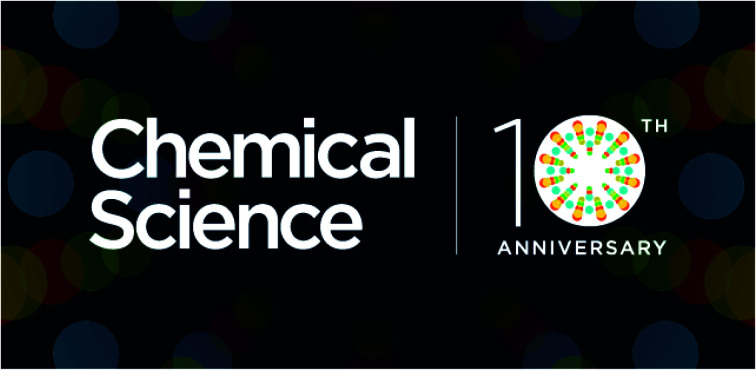

Welcome to the first of our special anniversary issues planned for this year, marking 10 years since *Chemical Science* published its first issue, back in July 2010.

We wanted to use these special birthday issues to recognise and thank members of our community who have been supporting the journal and publishing in *Chemical Science* since we launched ten years ago. So we have invited these authors to take part in these issues, by publishing their latest discoveries with us. Looking to the future, we have combined this with inviting a selection of researchers who are closer to the beginning of their careers, and who are new to publishing in *Chemical Science*. We hope that you enjoy reading this selection of articles, and that we can continue to provide an open and inclusive venue for both groups of researchers for many years to come.

This is also an opportunity to say thank you to all our reviewers of the journal and to our fantastic Editorial Board and Advisory Board members, both past and present, who have all contributed to the journal over the past ten years.

During the planning of these issues, looking back to 2010, we realised that one author, Chi-Ming Che from The University of Hong Kong, has published an article with us in every volume since the journal was launched. So we are delighted that he has contributed an article again for this first anniversary issue, and we are also taking this chance to showcase his work on our front cover.

We are sad that due to the current situation, we are unable to attend meetings and conferences this year to meet our authors in person. However in the spirit of this year, we wanted to take this opportunity to introduce to you to the *Chemical Science* Editorial team as it now looks in 2020.

We look forward to seeing you again in the near future, whether this is virtually or in person!

May Copsey, Executive Editor, and the *Chemical Science* Editorial team